# TgMORN1 Is a Key Organizer for the Basal Complex of *Toxoplasma gondii*

**DOI:** 10.1371/journal.ppat.1000754

**Published:** 2010-02-05

**Authors:** Aoife T. Heaslip, Florence Dzierszinski, Barry Stein, Ke Hu

**Affiliations:** 1 Department of Biology, Indiana University, Bloomington, Indiana, United States of America; 2 Institute of Parasitology, McGill University, Ste-Anne-de-Bellevue, Québec, Canada; Albert Einstein College of Medicine, United States of America

## Abstract

*Toxoplasma gondii* is a leading cause of congenital birth defects, as well as a cause for ocular and neurological diseases in humans. Its cytoskeleton is essential for parasite replication and invasion and contains many unique structures that are potential drug targets. Therefore, the biogenesis of the cytoskeletal structure of *T. gondii* is not only important for its pathogenesis, but also of interest to cell biology in general. Previously, we and others identified a new *T. gondii* cytoskeletal protein, TgMORN1, which is recruited to the basal complex at the very beginning of daughter formation. However, its function remained largely unknown. In this study, we generated a knock-out mutant of TgMORN1 (*ΔTgMORN1*) using a Cre-LoxP based approach. We found that the structure of the basal complex was grossly affected in *ΔTgMORN1* parasites, which also displayed defects in cytokinesis. Moreover, *ΔTgMORN1* parasites showed significant growth impairment *in vitro*, and this translated into greatly attenuated virulence in mice. Therefore, our results demonstrate that TgMORN1 is required for maintaining the structural integrity of the parasite posterior end, and provide direct evidence that cytoskeleton integrity is essential for parasite virulence and pathogenesis.

## Author Summary

The disease toxoplasmosis is the result of uncontrolled growth and proliferation of the intracellular parasite *Toxoplasma gondii*, which is pathogenic for most warm-blooded animals. If growth of the parasite is blocked, then it does not cause disease, even though it may persist in the host as a chronic infection. Proper assembly of the cytoskeleton of *T. gondii* is known to be essential for its growth, and consequently required for virulence. In this study, we investigated the function of a novel cytoskeletal protein, TgMORN1, in *T. gondii*. TgMORN1 is a major component of the basal complex, a novel cytoskeletal assembly located at the posterior end of the parasite. We found that TgMORN1 is required for maintaining the structural integrity of the parasite posterior end and is important for ensuring successful separation of daughters at late stage of parasite replication. In addition, infection with parasites deficient in TgMORN1 not only failed to kill mice but also provided protective immunity against a lethal challenge infection, indicating the importance of TgMORN1 in *T. gondii* growth both *in vitro* and *in vivo*.

## Introduction

*Toxoplasma gondii* is one of the most successful human parasites, infecting ∼30% of the total world population. It is the most common cause of congenital neurological defects in humans, and an agent for devastating opportunistic infections in immunocompromised patients. *T. gondii* is also a member of the phylum Apicomplexa, which contains thousands of species of obligate intracellular parasites [1]. Like *T. gondii*, many of these parasites (such as *Plasmodium spps*, the causative agents for malaria) pose serious health threats to human beings. The damage caused by these parasites absolutely depends on their ability to replicate. For instance, *T. gondii* causes severe lytic cerebral and ocular lesions when the immune system fails to control its proliferation. Massive proliferation of *Plasmodium* parasites often results in hemolytic anemia, parasite-mediated destruction of red blood cell; and cerebral malaria, caused by parasite-engorged erythrocytes clogging blood vessels in the brain [2]–[4]. An understanding of the growth and division of these parasites is therefore crucial for developing effective therapies. The *T. gondii* cytoskeleton provides the framework for organellar partitioning, maintains cell shape and drives invasion, thus is essential for parasite survival and proliferation. Furthermore, it is rich in structural features that are unique to the parasites, thus highly attractive potential drug targets for designing parasite specific drugs.

The cytoskeleton of *T. gondii* is complicated but highly ordered. Each parasite contains one cytoskeletal apical complex (made of 3 ring structures and 14 filaments of a novel tubulin polymer), and 22 cortical microtubules [5],[6]. Overlying the microtubules is a regular two-dimensional (2D) meshwork formed by intermediate-filament like proteins, subtended beneath a tri-layer membrane containing a highly ordered 2D array of intra-membranous particles [7]–[12]. Its actin cytoskeleton is extremely dynamic. Most of its actin are kept in the monomeric form, and only undergo very transient polymerization in extracellular parasites for driving parasite motility and invasion into the host cell [13]–[15]. The entire daughter cytoskeleton is assembled afresh in a reproducible temporal sequence within the mother during each round of parasite replication [12],[16],[17].

**Figure 1:**
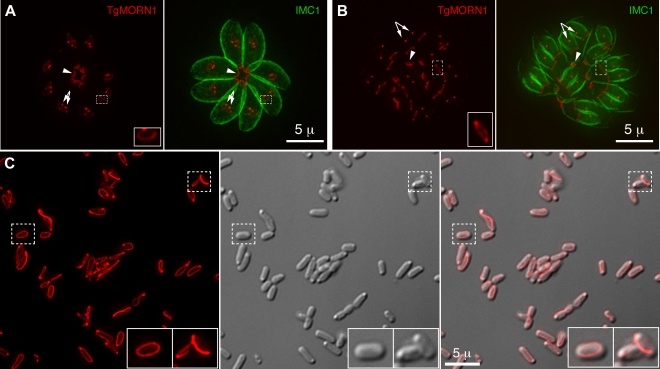
TgMORN1 is a component of the basal complex and forms rings and fibers when ectopically expressed in *E. coli*. **A–B**. The localization of TgMORN1 in parasites at the beginning (*A*) or late stage (*B*) of cell division. *Red*: eGFP-TgMORN1 pseudocolored red. *Green*: anti-IMC1 antibody labeling highlighting the protein network underneath the inner membrane complex (IMC). *Arrowheads*: the basal complexes of mother parasites. *Arrows*: spindle poles. Insets indicate the basal ring complexes of daughters and are at 2.5× magnification. **C**. 6XHIS-mCherryFP-TgMORN1 (*red*) expressed in *E. coli* formed rings and fibers. *Left*: Fluorescent image of mCherryFP-TgMORN1 containing rings and fibers. *Middle*: DIC image. *Right*: Overlay of the fluorescent image and the DIC. Insets are at 2× magnification.

Previously, we located a number of new cytoskeletal proteins, including TgMORN1 (Membrane Occupation and Recognition Nexus 1), TgCentrin2, and TgDLC- a member of the dynein light chain family, to a novel cytoskeletal structure at the extreme basal end of the parasite [18]–[20]. Due to its unique location and molecular composition, we named this structure “the basal complex” [18],[19]. We also discovered that although it will eventually become the extreme basal end of the parasite, the basal complex is initially constructed at the very beginning of daughter formation (Figure 1A), and in juxtaposition to the future apical end [18],[19]. It first appears as a TgMORN1-containing ring that caps the growing ends of the daughter cortical cytoskeletons. The cap is maintained throughout daughter development (Figure 1B), and eventually constricts into a cone at the basal end of the parasite when the daughter parasite becomes mature. It was a great surprise to find that the basal complex is formed so early, and equally surprising that it is initiated at the same site where the very first elements of the future apical complex are laid down. This highly suggestive combination of unexpected timing and unexpected location prompted the hypothesis that components of the basal complex might play some unknown critical role in the assembly of the daughter cortical cytoskeleton.

To address the role of the basal complex in *T. gondii* physiology, we decided to dissect the function of TgMORN1, as it is a major basal complex component and is also the earliest basal complex component identified so far. We found that TgMORN1 formed rings and fibers when ectopically expressed in bacteria, supporting the notion that it might function as a structural protein. We also made a knock-out mutant of TgMORN1 (*ΔTgMORN1*) and discovered that the structure of the parasite posterior end was grossly altered upon the loss of TgMORN1. Interestingly, *ΔTgMORN1* parasites displayed cytokinesis defects, apicoplast segregation defects and growth defects *in vitro*. In mice, these parasites not only were avirulent but also provided protective immunity against a lethal challenge infection.

## Results

### TgMORN1 forms rings and fibers when ectopically expressed in *E. coli*

The MORN-domain is a structural module conserved from bacteria to human. MORN-domain containing proteins have been found in large protein complexes and are thought to mediate protein-protein or protein-lipid interactions [21]–[25]. TgMORN1 is mainly formed of 14 MORN repeats [18]–[20]. When ectopically expressed in *E. coli*, 6XHIS-mCherryFP-TgMORN1 was assembled into rings and fibers in the absence of other *T. gondii* proteins (Figure 1C), suggesting that the tendency of TgMORN1 to self-assemble might be involved in forming the basal ring structure during daughter construction (Figure 1A). 6XHIS-TgMORN1 and 6XHIS-eGFP-TgMORN1 formed similar structures when expressed in *E. coli*, with higher tendency to form fibers (data not shown).

### Generation of TgMORN1 knockout parasites using Cre-LoxP based strategy

**Figure 2:**
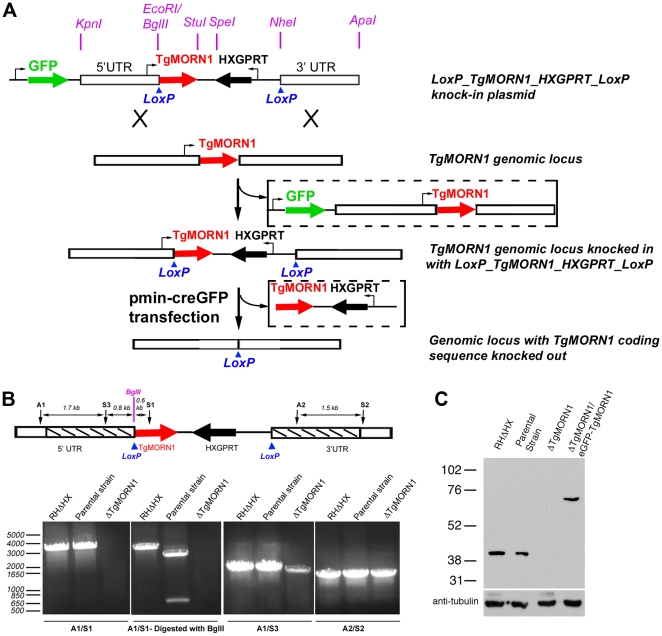
The generation of TgMORN1 knockout (*ΔTgMORN1*) parasites. **A**. Diagram describing the procedure for generating the *ΔTgMORN1* parasite line. See text for details. **B**. Genomic PCR analysis of RHΔHX, the parental (*LoxP-TgMORN1-HXGPRT-LoxP*), and *ΔTgMORN1* parasites. The diagram at the top shows the positions of the sequences that the PCR primers (A1, A2, S1, S2, S3) hybridize with. Boxes marked by slanted lines indicate regions of 5′ and 3′ UTR of TgMORN1 gene included in LoxP_TgMORN1_HXGPRT_LoxP knock-in plasmid (*c.f.* *Figure 2A*). **C**. Western blot analysis of RHΔHX, parental, *ΔTgMORN1*, and the complemented (*ΔTgMORN1/eGFP-TgMORN1*) parasites, which shows that the level of TgMORN1 expression was comparable among RHΔHX, the parental strain and the complemented parasites, but was undetectable in *ΔTgMORN1* parasites. The blot was reprobed with mouse-anti-tubulin B-5-1-2 to use α-tubulin as a loading control.

To further understand its function, we decided to generate a knockout mutant of TgMORN1. Several attempts to eliminate TgMORN1 expression by one-step homologous recombination in RHΔHXGPRT (RHΔHX) parasites failed (unpublished results). These failures were not due to low homologous recombination frequency, as a parasite line in which endogenous TgMORN1 gene was replaced by homologous recombination with “LoxP-TgMORN1-HXGPRT-LoxP” was obtained fairly easily in the same set of experiments (one out of nine clones screened was positive) (Figure 2A), suggesting that the loss of TgMORN1 confers serious growth disadvantage to the parasite. In the “LoxP-TgMORN1-HXGPRT-LoxP” parasite line, the TgMORN1 coding sequence was flanked by two LoxP sites, allowing for the excision of the [TgMORN1-(HXGPRT expression cassette)] fragment after the transient transfection of a plasmid expressing Cre-eGFP into the parasite (Figure 2A). 6-thioxanthine (6-TX) selection was then applied to select for parasites that had lost HXGPRT activity, and five TgMORN1 negative clones (out of total 41 clones screened by immunofluorescence using a rat anti-TgMORN1 antibody) were obtained. Genomic PCR and western blot analysis confirmed the disruption of the *TgMORN1* locus and the complete loss of TgMORN1 protein expression in the TgMORN1 knockout parasite (*ΔTgMORN1*) (Figure 2B&C).

### The loss of TgMORN1 affects the organization of the parasite posterior end

**Figure 3:**
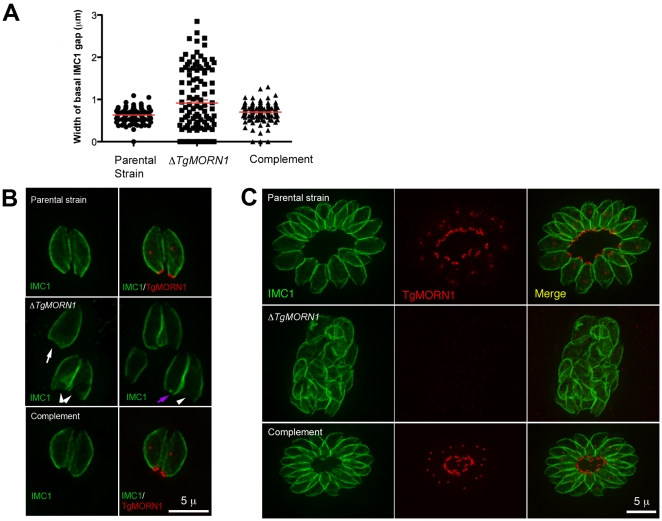
The loss of TgMORN1 affects the organization of the parasite posterior end. **A**. The comparison of the distribution of the width of basal IMC1 gap in the parental strain (n = 102), *ΔTgMORN1* (n = 114), and the complemented - *ΔTgMORN1/eGFP-TgMORN1* parasites (n = 118). Red lines indicate the average. **B**. The comparison of parasite morphology of the parental strain (*top panels*), *ΔTgMORN1* (*middle panels*), and the complemented (*bottom panels*) parasites in vacuoles containing one or two parasites. Notice the heterogeneity of the posterior end morphology in *ΔTgMORN1* parasites. Three main classes of morphology are shown: parasites with wide basal IMC1 gap (*white arrowheads*), parasites with small or no basal IMC1 gap but wide posterior end (*white arrow*), and parasites with a largely normal posterior end that sometimes contained irregular IMC structure (*purple arrow*). *Green*: anti-IMC1 antibody labeling. *Red*: anti-TgMORN1 labeling (the parental strain) or eGFP-TgMORN1 labeling (the complement). **C**. The comparison of parasite morphology of the parental strain (*top panels*), *ΔTgMORN1* (*middle panels*), and the complemented (*bottom panels*) parasites in vacuoles ≥16 parasites. Notice the highly irregular organization of *ΔTgMORN1* parasites within the vacuole. *Green*: anti-IMC1 antibody labeling. *Red*: anti-TgMORN1 labeling (the parental strain, *ΔTgMORN1* parasites) or eGFP-TgMORN1 labeling (the complement).

It was immediately noticeable that the posterior ends of the *ΔTgMORN1* parasites were highly irregular and heterogeneous and that the distribution of the width of IMC1 basal gap among these parasites was much more wide-spread than those of the parental strain (*LoxP-TgMORN1-HXGPRT-LoxP*) and the complement (*ΔTgMORN1/eGFP-TgMORN1*) (Figure 3A). The morphologies of *ΔTgMORN1* parasites posterior ends can be clustered into three major groups. About 37% of the parasites had a basal IMC1 gap that was more than 1 µm (Figure 3B; white arrowheads), much wider than the average of the basal IMC1 gap of the parental strain, which was ∼0.65 µm. In the second group (∼35%), the basal IMC1 gap was either small or nonexistent, but the posterior ends of the parasites still appeared to be significantly wider than that of the parental strain, giving the parasite a “triangular” shape (Figure 3B; white arrow). In the third group (∼28%), the width of the parasite posterior end looked normal, however, irregular basal IMC structure was often present (Figure 3B; purple arrow). Likely a direct result of distorted parasite shape, *ΔTgMORN1* parasites never formed a “rosette”- a common organization of wild-type parasites in vacuoles containing more than 8 parasites (Figure 3C). These defects were fully corrected in the *ΔTgMORN1/eGFP-TgMORN1* line (Figure 3A–C). The arrangement of cortical microtubules around *ΔTgMORN1* parasite cortex appeared to be normal (Figure S1).

**Figure 4:**
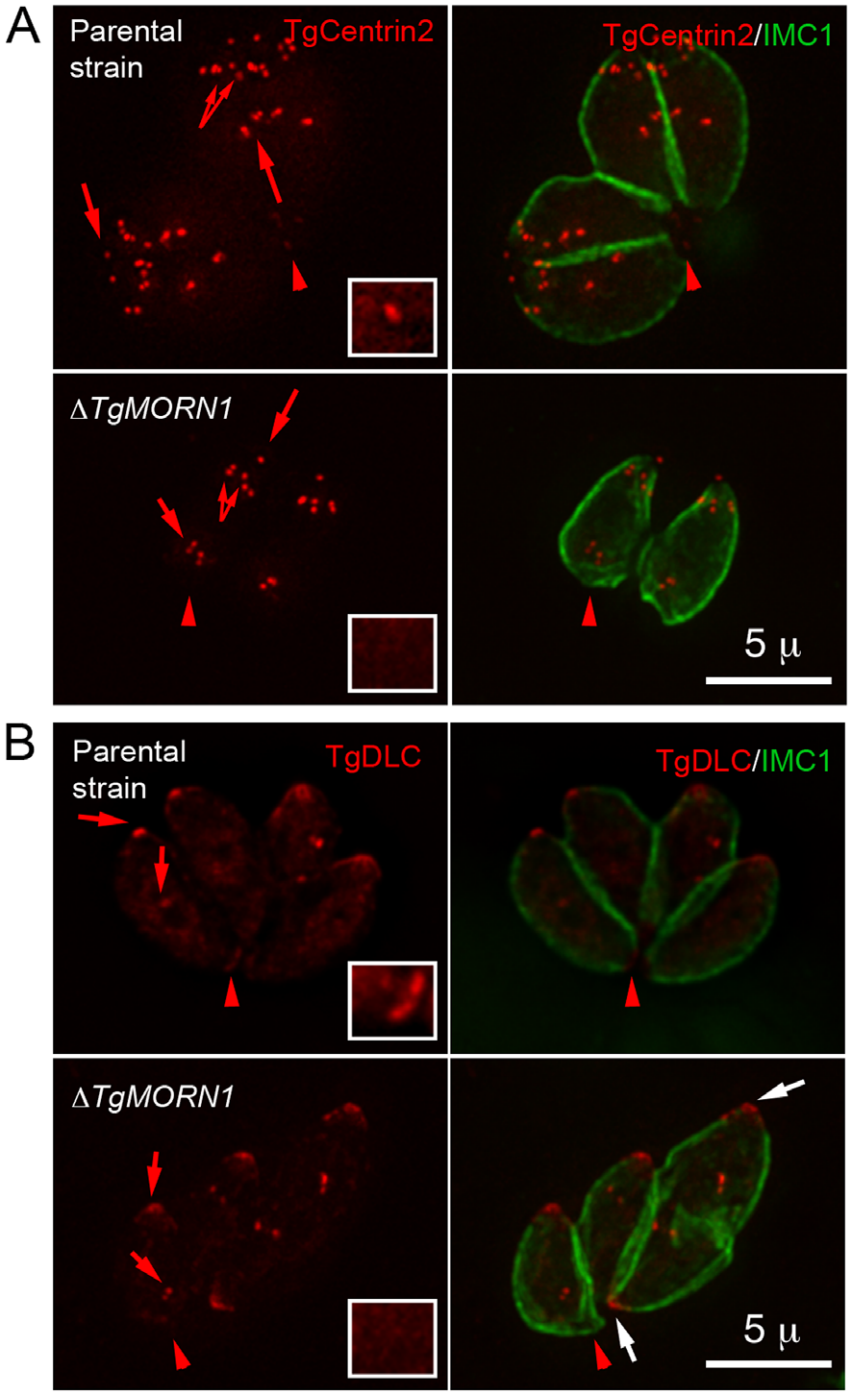
The loss of TgMORN1 affects the localization of other proteins to the basal complex. **A**. When eGFP-TgCentrin2 (pseudo-colored red) was expressed in *ΔTgMORN1* parasites (*bottom panels*), it was incorporated into the apical complex, the centrioles and the peripheral annuli (*arrows*) as in the parental strain (*top panels*), but eGFP-TgCentrin2 basal complex localization (*arrowheads*, *insets*) was undetectable in these parasites. Insets are at 2× magnification and contrast enhanced to visualize the basal eGFP-TgCentrin2 structure. **B**. When eGFP-TgDLC (pseudo-colored red) was expressed in *ΔTgMORN1* parasites (*bottom panels*), it was incorporated into the apical complex, and spindle pole/ centriole assembly (*red arrows*) as in the parental strain (*top panels*), but an eGFP-TgDLC concentration could not be detected in the parasite posterior end (*red arrowheads*, *insets*). *White arrows* indicate the apical complexes of two parasites that failed to complete cytokinesis in the previous round of replication. Insets are at 2× magnification and contrast enhanced to visualize the basal eGFP-TgDLC structure.

As TgMORN1 is the earliest component recruited to the basal complex identified so far, we examined how the loss of TgMORN1 affected the localization of several other basal complex components. In *ΔTgMORN1* parasites, eGFP-TgCentrin2 basal complex localization was undetectable, although the localization of eGFP-TgCentrin2 to the apical complex, the centrioles and the peripheral annuli was not affected (Figure 4A). Similarly, when eGFP-TgDLC was expressed in *ΔTgMORN1* parasites, it was incorporated into the apical complex and spindle pole/centriole assembly as in the parental strain, but an eGFP-TgDLC concentration could not be detected at the parasite posterior end (Figure 4B). There could be two plausible explanations for the undetectability of an eGFP-TgCentrin2 or TgDLC basal concentration in *ΔTgMORN1* parasites. It is possible that these two proteins failed to be recruited to the parasite basal complex because of the loss of TgMORN1. Alternatively, these proteins could spread to larger area in the disorganized parasite posterior end, which would dilute the signal, rendering it undetectable.

### The loss of TgMORN1 has little effect in invasion

**Figure 5:**
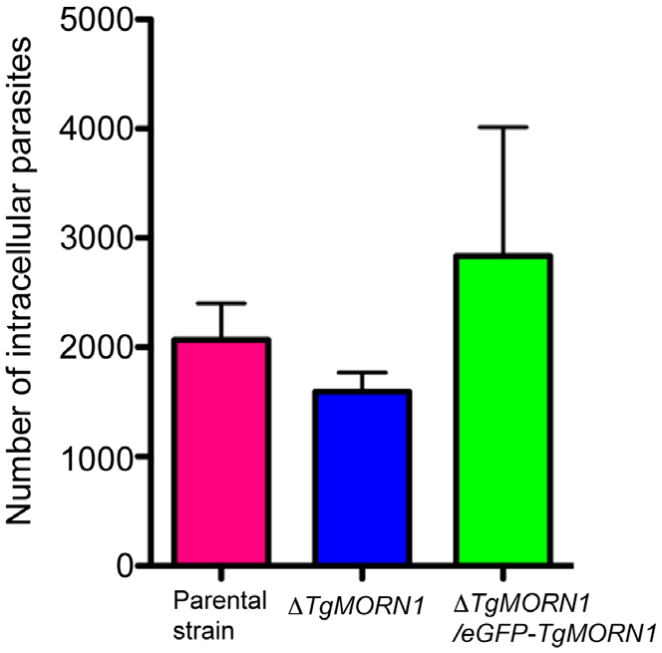
The loss of TgMORN1 has little effect in invasion. Graph shows the number of parasites invaded after equal number of extracellular parental, *ΔTgMORN1* or *ΔTgMORN1/eGFP-TgMORN1* parasites were added to confluent HFF monolayers and incubated at 37°C for 1 hour. Result is the summary of three independent experiments. Error bar: standard error of the mean. P values for the comparison between *ΔTgMORN1* and parental parasites; and *ΔTgMORN1* and complemented parasites are 0.27 and 0.35 respectively (Student t-test).

To assess the defects in parasite invasion in *ΔTgMORN1* parasites, we performed invasion and gliding motility assays. We did not detect a significant difference in invasion among the parental, *ΔTgMORN1* and the complemented parasites (Figure 5). P values for the comparison between *ΔTgMORN1* and parental parasites; and *ΔTgMORN1* and complemented parasites were 0.27 and 0.35 respectively.). We also did not observe qualitative differences among the trails deposited by the parental, *ΔTgMORN1* and the complemented parasites in gliding motility assays (data not shown).

### *ΔTgMORN1* parasites display defects in cytokinesis and apicoplast segregation

**Figure 6:**
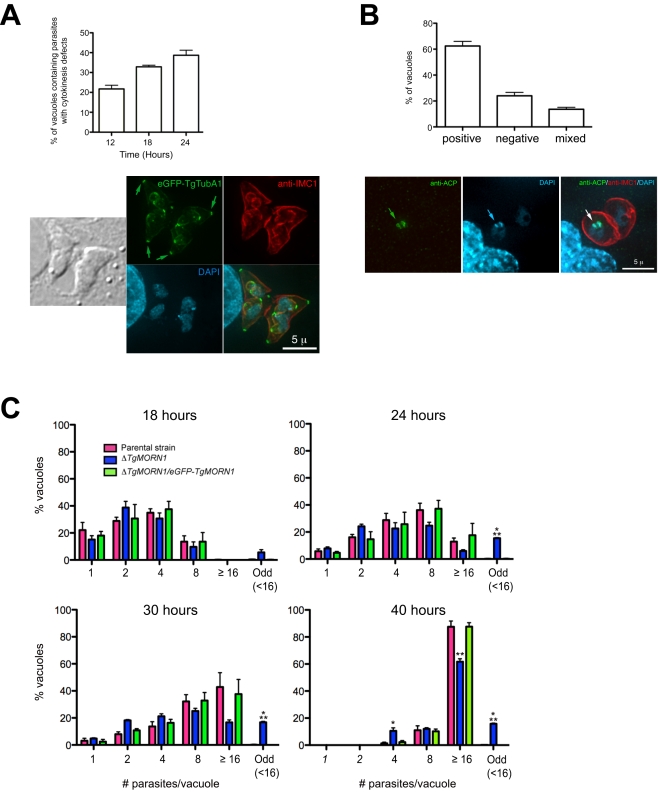
*ΔTgMORN1* parasites display defects in cytokinesis and apicoplast segregation. **A**. *Top*: Graph shows the percentages of vacuoles with *ΔTgMORN1* parasites displaying cytokinesis defects at 12, 18, 24 hours post infection. Error bar: standard error of the mean. *Bottom*: Images show a parasitophorous vacuole containing two pairs of *ΔTgMORN1* parasites, both of which have failed cytokinesis in the previous round of cell division. These parasites expressed eGFP-TgTubA1 (*green*) and were also labeled with anti-IMC1 (*red*), showing that the apical complex, cortical microtubules and IMC1 network all formed but the parasites failed to separate before beginning the next round of daughter construction. Arrows indicate the apical complexes of the mother parasites. **B**. *Top*: Graph shows the percentages of vacuoles with *ΔTgMORN1* parasites scored as apicoplast positive (*i.e.* all parasites in the vacuole contain apicoplast, ∼62%), apicoplast negative (*i.e.* none of the parasites in the vacuole contains apicoplast, ∼24%) or mixed (*i.e.* vacuoles contains both apicoplast positive and negative parasites, ∼14%) (n = 250). Error bar: standard error of the mean. No apicoplast negative parasites were observed in either the parental or complemented strains (n = 50). *Bottom*: Images show a parasitophorous vacuole containing two *ΔTgMORN1* parasites, where one contains apicoplast (arrows) and the other one does not. *Green*: anti-ACP; *red*: anti-IMC1; *cyan*: DAPI. **C**. Comparison of the intracellular growth of *ΔTgMORN1* parasites with those of the parental strain and *ΔTgMORN1/eGFP-TgMORN1* parasites 18, 24, 30 and 40 hours after infection. The trend shows a decrease in replication rate upon the loss of TgMORN1. It also shows that asynchronized replication within the parasitophorous vacuole occurred much more frequently in *ΔTgMORN1* parasites than in the parental strain and *ΔTgMORN1/eGFP-TgMORN1* parasites. “Odd” group includes vacuoles in which the number of parasites was less than 16, and not an integral power of 2. “≥16” group includes all the vacuoles that contained 16 or more parasites. Error bar: standard error of the mean. “*” indicates P values less than 0.05, “**” indicates P values less than 0.01, and “***” indicates P values less than 0.0001 (Student t-test), when *ΔTgMORN1* parasites are compared with the parental strain or *ΔTgMORN1/eGFP-TgMORN1* parasites. For the 40-hour time point, vacuoles containing one and two parasites were not included in the counting, as they were most likely secondary vacuoles formed by parasites that egressed from large primary vacuoles and reinvaded.

To assess the defects in parasite replication in *ΔTgMORN1* parasites, we examined their intracellular growth (Figure 6). We found that a significant percentage of vacuoles contained parasites displaying cytokinesis defects, where daughter parasites failed to separate after budding (*c.f.* Figure 4B). This percentage increased from 21% (12 hours post infection) to 38% (24 hours post infection) as the total number of cell division events increased over time (Figure 6A, top). For the parental strain or *ΔTgMORN1/eGFP-TgMORN1* parasites, less than 0.5% of the total vacuoles contained parasites displaying similar defects. The construction of new daughter parasites was observed in parasites where cytokinesis had failed (Figure 6A, bottom), suggesting that the completion of cytokinesis and the initiation of the next round of daughter formation are not tightly coupled, confirming previous observations [12],[26],[27].

To examine if *ΔTgMORN1* parasites are defective in organelle biogenesis, we examined the segregation of the apicoplast, the mitochondrion as well as the *de novo* formation of the secretory organelles: dense granules, micronemes, and rhoptries. While we did not detect any significant defects in the segregation or synthesis of other organelles in these parasites, we found that parasites in ∼24% of the vacuoles contained no apicoplast (revealed by an antibody recognizing the Acyl Carrier Protein (ACP), an apicoplast protein [28]), indicating a modest apicoplast segregation defect (n = 250, Figure 6B). No apicoplast negative parasites were observed in either the parental or complemented parasites (n = 50).

When numbers of parasites per vacuoles were counted for 18, 24, 30 and 40 hours post-infection, we found a trend showing a decrease in replication rate upon the loss of TgMORN1 (Figure 6C). This assay also showed that asynchronous replication within the parasitophorous vacuole occurred much more frequently in *ΔTgMORN1* parasites, where ∼15% of vacuoles at 24–40 hours post-infection, contained “odd” number (≠ 2^n^ and <16) of parasites, comparing with less than 0.4% for the parental strain and *ΔTgMORN1/eGFP-TgMORN1* parasites (Figure 6C).

### *ΔTgMORN1* parasites display growth defects *in vitro* and greatly attenuated virulence in mice

**Figure 7:**
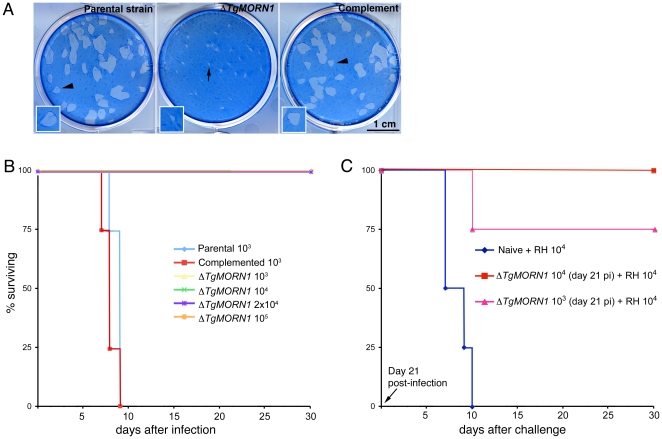
*ΔTgMORN1* parasites display growth defects *in vitro* and greatly attenuated virulence in mice. **A**. *ΔTgMORN1* parasites formed plaques (*middle*, *arrow*) significantly smaller than those formed by the parental (*left*), and the complemented (*ΔTgMORN1/eGFP-TgMORN1*) strains (*right*) (*arrowheads*). Insets are at 2× magnification. **B**. CD1 outbred mice were infected intraperitoneally with either 10^3^ or 10^4^ parental; 10^3^, 10^4^, 2×10^4^ or 10^5^ *ΔTgMORN1*; or 10^3^ or 10^4^ *ΔTgMORN1/eGFP-TgMORN1* parasites. Mice that were infected with parental parasites died between day 7 and day 9 pi. In contrast, mice that were challenged with 10^3^, 10^4^, 2×10^4^ or 10^5^ *ΔTgMORN1* parasites remained alive. EGFP-TgMORN1 complementation restored parasite virulence. For the parental and *ΔTgMORN1/eGFP-TgMORN1* parasites, only the data for 10^3^ parasites infection is shown. All surviving mice remain healthy for more than 60 days after infection. Data for only the first 30 days of the experiment is shown. **C**. At day 21 pi, surviving mice “immunized” with 10^3^ or 10^4^ *ΔTgMORN1* parasites were challenged with 10,000 wild type RH parasites (LD_100_ = 1). Compared to naïve mice, which died between day 7 and day 10 pi, mice that were pre-infected with *ΔTgMORN1* parasites were protected from lethal challenge, where all mice immunized with 10^4^ *ΔTgMORN1* parasites and 75% of mice immunized with 10^3^ *ΔTgMORN1* parasites remained alive. All surviving mice remain healthy for more than 60 days after the challenge. Data for only the first 30 days of the experiment is shown.

To evaluate how the infectivity of the parasite was affected by TgMORN1 deficiency, we performed plaque assays and found that *ΔTgMORN1* parasites formed plaques significantly smaller than those formed by the parental and *ΔTgMORN1/eGFP-TgMORN1* parasites (Figure 7A). Because of this growth defect observed *in vitro*, the effect of TgMORN1 deletion on parasite virulence in mice was investigated. CD1 outbred mice were infected intraperitoneally with either 10^3^ or 10^4^ parental; 10^3^, 10^4^, 2×10^4^ or 10^5^ *ΔTgMORN1*; or 10^3^ or 10^4^ *ΔTgMORN1/EGFP-TgMORN1* tachyzoites (Figure 7B). Mice that were infected with parental parasites started to show signs of disease (*i.e.* ascites due to tachyzoites in the peritoneum, ruffled fur) at day 5 post-infection (pi), and died between day 7 and day 9 pi, as expected. In contrast, mice that were challenged with 10^3^ or 10^4^ *ΔTgMORN1* parasites showed no signs of disease and remained alive. Mice that received 2×10^4^ or 10^5^ *ΔTgMORN1* tachyzoites showed a slightly swollen abdomen as sole sign of disease and remained alive. EGFP-TgMORN1 complementation restored parasite virulence, as mice that were infected with complemented parasites followed the same pattern as mice that were infected with the parental strain (Figure 7B). At day 21 pi, surviving mice “immunized” with 10^3^ or 10^4^ *ΔTgMORN1* parasites were challenged with 10,000 wild type RH tachyzoites (LD_100_ = 1). Compared to naïve mice, which died between day 7 and day 10 pi, mice that were pre-infected with *ΔTgMORN1* parasites were protected from lethal challenge, where all mice immunized with 10^4^ *ΔTgMORN1* parasites and 75% of mice immunized with 10^3^ *ΔTgMORN1* parasites remained alive and healthy more than 60 days after the challenge (Figure 7C).

## Discussion

### The Cre-LoxP based knock-out strategy

Several knock-out strategies have been developed to study protein function in *T. gondii*. One-step homologous replacement [29] has been used to study the function of many non-essential genes. For single-copy essential genes, however, no viable knock-out mutant strain can be obtained using this method, because *T. gondii* grown in lab culture is asexual and contains a haploid genome. In fact, for any gene the loss of which results in ∼20% reduction in growth rate per cell cycle, it is practically impossible to acquire a knockout mutant using this method, as after ∼21 cell cycles (i.e. ∼7 days, the usual timeframe for drug selection before cloning), the percentage of the knockout parasite in the population will drop to less than ∼0.03% (0.8^21^×0.03; 0.03 is the ratio of homologous vs nonhomologous events estimated in [29]). The failure to generate TgMORN1 knock-out mutants using one-step homologous replacement led us to implement the Cre-LoxP recombination technique [30],[31] (Personal Communications, Drs Gusti Zeiner, Michael Reese and John Broothroyd at Stanford University). There are two potential advantages of this strategy compared with the previously developed methods [29],[32]. First of all, it can be used for generating knock-out for genes whose function is sensitive to its expression level, because in the “LoxP-GENEX-HXGPRT-LoxP” intermediate, the expression of the target gene is driven by its endogenous promoter. Secondly, this strategy makes it easier to obtain a clonal population of knock-out mutants that have severe growth defects, because the loss of the target gene via recombination by Cre recombinase simultaneously results in the loss of HXGPRT, which allows for the enrichment of the knockout mutants through a subsequent 6-TX selection that highly inhibits the growth of parasites where recombination has not occurred.

### The function of TgMORN1

The initiation, construction and the maturation of different parts of *T. gondii* cytoskeleton occur in a highly reproducible sequence. We have previously demonstrated that the basal complex was assembled at the beginning of daughter construction, prompting our hypothesis that the basal complex might play a guiding role in the initiation of the parasite cortical cytoskeleton [18],[19]. We were surprised to see, however, that although TgMORN1 is a prominent component of the basal complex, *ΔTgMORN1* parasites managed to construct a cortical cytoskeleton functional enough to support parasite survival *in vitro*. This indicates that if the basal complex dictates the initiation of the daughter cortical cytoskeleton, it involves basal complex components other than TgMORN1. The prediction is that if such protein exists, its recruitment to the basal complex should be early and independent of TgMORN1.

Once the daughters are constructed, they need to be properly segregated to become a functional entity. We have previously proposed that the basal complex might play a role in this maturation step based on the correlation between the timing of the basal complex constriction and that of cytokinesis initiation [19]. The phenotypes of *ΔTgMORN1* parasites support this hypothesis. The architecture of the posterior end in these parasites was clearly perturbed, which correlated with a significant increase in defective cytokinesis. This might be a direct result of losing TgMORN1, a major structural protein in the basal complex. Alternatively, the indirect effect on the localization of other basal complex components, such as TgCentrin2, might also play a role in producing this structural defect, as we found previously that TgCentrin2 containing basal structure underwent contraction when the intracellular calcium concentration was elevated [19]. We do not know at this time if TgMORN1 itself plays an active role in recruiting these basal complex components or if the basal structure formed by TgMORN1 provides a necessary platform for protein association. These defects in the biogenesis of the basal complex are likely the direct cause for the inefficient daughter parasite separation in *ΔTgMORN1* parasites. Moreover, TgMORN1 most likely works with other proteins in the basal complex to drive cytokinesis, as the “penetrance” of the cytokinesis defect was not complete in the TgMORN1 deficient parasites. Besides cytokinesis defects, *ΔTgMORN1* parasites displayed a modest apicoplast segregation defect, which also likely contributed to the growth defects of these parasites, as “apicoplast-less” parasites were shown to have a “delayed-death” phenotype [33]–[35]. It was proposed that the apicoplast is anchored to the centrosome during apicoplast division based on the close proximity between these two organelles [36],[37]. It will be interesting to elucidate in the future whether a direct connection between these two organelles exists and if TgMORN1 plays a role in the formation/maintenance of such a connection, or the apicoplast segregation defect in *ΔTgMORN1* parasites is an indirect result of other defects in these parasites.

In sum, we have implemented a new knock-out strategy that is generally applicable to studying the functions of genes that are important for parasite growth. In addition, *ΔTgMORN1* mutant will provide a convenient background to generate multi-gene knockouts for systematic dissection of the function of the basal complex and the interaction among its proteins. It will also facilitate mutagenesis analysis to understand the structural role of the MORN domain in general. Finally, our study provides direct evidence that cytoskeleton integrity is essential for parasite virulence and pathogenesis, as TgMORN1 deficiency has a profound effect on parasite virulence *in vivo*.

## Materials and Methods

### Ethics statement

All mice were maintained under specific-pathogen-free conditions in accordance with institutional guidelines of Institute of Parasitology, McGill University, Ste-Anne-de-Bellevue, H9X3V9, QC, Canada. The animal protocol was approved by the McGill University Macdonald campus Facility Animal Care Committee.

### Plasmids construction

The constructs for generating the parental strain: “LoxP-TgMORN1-HXGPRT-LoxP” parasite were produced using PTKO2_II as the backbone, and PTKO2_II was constructed based on PTKO (a kind gift from Drs Gusti Zeiner, Michael Reese and John Broothroyd at Stanford University). PTKO contains total two LoxP sites and two multiple cloning sites (MCS), with one MCS (MCS1) placed at the 5′ end of the first LoxP site and the second MCS (MCS2) placed at the 3′ end of the second LoxP site. In PTKO2_II, an additional MCS (MCS3) (AGATCTGTTTAAACGCGATCGCGGTCCGAGGCCT) was added to the 3′ end of the first LoxP site in PTKO, and 3′ portion of MCS1 in PTKO was modified to (GGTACCCTCGAGGATATCTACGAATTC). To construct PTKO2_II, a 521bp DNA fragment (Table S1) containing these changes was synthesized and cloned into PUC-57 (Genscript, Inc, Piscataway, NJ), digested with KpnI and SpeI, and ligated into PTKO to replace the corresponding KpnI-SpeI fragment on PTKO. To construct the “LoxP-TgMORN1-HXGPRT-LoxP” plasmid, first, a 2.1kb fragment located to the 3′ end of TgMORN1 coding sequence in the genome was amplified using HK180 and HK181 (Table S1), digested with NheI and ApaI and ligated into NheI and ApaI sites of PTKO2_II (*c.f.* Figure 2A), which resulted in the plasmid PTKO2_II_3′UTR. Secondly, a ∼2.3kb fragment located to the 5′ end of TgMORN1 coding sequence in the genome was amplified using primers HK182 and HK183 (Table S1), digested with EcoRI and KpnI, and ligated into EcoRI and KpnI sites of PTKO2_II_3′UTR (*c.f.* Figure 2A), which resulted in the plasmid PTKO2_II_TgMORN1_5_3′UTR. Lastly, TgMORN1 coding sequence was amplified from pmin-eGFP-TgMORN1 [18] using primers HK191 and HK193 (15 bp of TgMORN1 Kozak sequence was included in the primer HK191) (Table S1), digested with BglII and StuI and ligated into BglII and StuI sites (*c.f.* Figure 2A) of PTKO2_II_TgMORN1_5_3′UTR to give the “LoxP-TgMORN1-HXGPRT-LoxP” plasmid. Genomic DNA for amplifying the 5′ and 3′ UTR of TgMORN1 was harvested from RH parasites using Qiagen DNeasy Blood & Tissue kit (Cat# 69504, Qiagen).

ptub-Cre-GFP was generated by amplifying Cre-GFP from pCAG-Cre:GFP (Plasmid 13776, Addgene) using primers HK223 and HK224 (Table S1). The PCR product was then digested with NheI and AflII and ligated immediately downstream of the ptub promoter in place of mCherryFP-eGFP/ NheI-AflII in ptub-mCherryFP-eGFP (a kind gift from Dr. John Murray, University of Pennsylvania). pmin-Cre-GFP was constructed by replacing eGFP-TgDLC/NheI-AflII in pmin-eGFP-TgDLC [18] with Cre-GFP/NheI-AflII from ptub-Cre-GFP.

To construct pQE30-6xHIS-TgMORN1, TgMORN1/BglII-AflII from pmin-eGFP-TgMORN1 was ligated into pQE30-DIP13_AflII [38] to replace DIP13/BamHI-AflII. To construct pQE30-6xHIS-mCherryFP-TgMORN1, mCherryFP-TgMORN1/NheI-AflII from pmin-mCherryFP-TgMORN1 [19] was ligated into PQE30-DIP13_AflII_NheI [38] to replace DIP13/NheI_AflII. The construction of pQE30-6xHIS-TgMORN1 and pQE30-6xHIS-mCherryFP-TgMORN1 was carried out by Biomeans Inc. (Sugar Land, TX).

### Expression of 6XHIS-mCherryFP-TgMORN1 in *E. coli* and sample preparation for imaging

Frozen stocks of BL21(DE3)pLysS bacteria transformed with pQE30-6XHIS-mCherryFP-TgMORN1 were made from cultures of single colonies. The frozen stock were then streaked on LB agar plates containing 100µg/ml of ampicillin, 50µg/ml chloramphenicol and grown at 37°C for 24 hours, then at 4°C for ∼24–48 hours. Alternatively, liquid cultures were grown from frozen stocks at 37°C in 100 ml LB containing 100µg/ml of ampicillin, 50µg/ml chloramphenicol (LB-amp-cap) for ∼24 hours. For unknown reasons, 6XHIS-mCherryFP-TgMORN1 expression in *E. coli* varied among cultures when grown in suspension and in a given experiment usually about 1 in 3 cultures expressed 6XHIS-mCherryFP-TgMORN1 well, forming rings and fibers in ∼100% of the bacteria. The expression of 6XHIS-mCherryFP-TgMORN1 in bacteria grown on LB agar plate was more consistent. Samples were processed as described in [39] before imaging.

### Parasite culture and transfection

*T. gondii* tachyzoites were used in all experiments, and the maintenance of parasites by continuous passage in human foreskin fibroblasts (HFFs) and parasite transfections were performed as previously described [40].

### Generation of *ΔTgMORN1* and *ΔTgMORN1/eGFP-TgMORN1* parasites

33 µg LoxP-TgMORN1-HXGPRT-LoxP plasmid was linearized with ApaI (*c.f.* Figure 2A) and transfected into ∼1×10^7^ RHΔHXGPRT (RHΔHX) parasites [41], and selected by MPA (25µg/ml) and xanthine (50µg/ml). GFP negative parasites were collected by flow cytometry and 3 parasites were sorted into each well of a 96 well plates containing HFF. Single clones were then amplified, and screened by PCR for parasites where the endogenous TgMORN1 locus has been replaced by LoxP-TgMORN1-(HXGPRT expression cassette)-LoxP. This parasite line, named LoxP-TgMORN1-HXGPRT-LoxP /parental strain was then transfected with 25 µg pmin-Cre-GFP plasmid to excise the fragment between LoxP, and then placed under 80µg/ml 6-thioxanthine (6-TX) selection. Single clones were chosen after the second and third passage of the 6-TX resistant population, and TgMORN1 knockout (*ΔTgMORN1*) parasites were first selected by immunofluorescence using a rat anti-TgMORN1 antibody and subsequently confirmed by genomic PCR and western blotting.

To generate *ΔTgMORN1/eGFP-TgMORN1* parasites, 7×10^6^ *ΔTgMORN1* parasites were transfected with 25µg pmin-eGFP-TgMORN1 plasmid [18] and grown without applying any drug selection. After ∼16 days, 100% of the parasites expressed eGFP-TgMORN1, which were then used for assessing the effectiveness of the complementation. pmin-eGFP-TgMORN1 plasmid contains no *T. gondii* selectable marker. Therefore like *ΔTgMORN1* parasites, *ΔTgMORN1/eGFP-TgMORN1* parasites were also HXGPRT deficient.

### Expression and purification of 6XHIS-TgMORN1

Single BL21(DE3)pLysS bacterial colonies containing pQE30-6xHIS-TgMORN1 plasmid were grown overnight in LB-amp-cap at 37°C. Cultures were then diluted 1∶20 in 2 liter LB-amp-cap and grown till OD_600_ reached ∼0.6–0.8 before the addition of isopropyl β-D-1-thiogalactopyranoside (IPTG) to 1 mM. Cultures were then grown for additional 4 hours at 37°C. Cells were then pelleted at 6,000 rpm for 25 minutes, resuspended in 40 ml of cold lysis buffer (8 mM Tris-Ac pH 7.5, 3 mM Trisbase, 100 mM KAc, 1 mM MgAc) containing 9.6g of cell lytic express (∼8 vials, Cat# C1990, Sigma), 1 µM TAME (Cat# T4626, Sigma) and 1 µM PMSF (Cat# P7626, Sigma), and incubated at 4°C for ∼60 minutes. Cells were sonicated∼five times for 30 seconds each with 1 minute cooling between each cycle, then centrifuged at 15,000 rpm for 15 minutes at 4°C. 1.9 ml packed Talon resin (Cat# 635501, Clontech) equilibrated with lysis buffer was then added to the supernatant and gently mixed at 4°C for 1 hour. The resin was then washed with lysis buffer with 10mM imidazole 4 times and eluted with ∼1.5 ml 1XLDS sample buffer and reducing reagents buffer (Cat# NP0007, Invitrogen).

### Generation and affinity purification of TgMORN1 antibody

Eluted 6XHIS-TgMORN1 proteins (see above) were loaded on 4–12% Bis-Tris gel and gel slices containing 6XHIS-TgMORN1 were then used to inject rats for antibody production (Cocalico Biological, Inc). The affinity purification of TgMORN1 antibody was carried out as described in [42]. Briefly, PVDF membrane blot with immobilized recombinant 6XHIS-TgMORN1 was blocked in 1% “Blotto”(Cat# 1152709001, Roche) in TBS (20mM Tris base pH 7.4, 150mM NaCl) for 30min; diluted rat anti-TgMORN1 serum (1∶10, diluted in 0.5%“Blotto” in 1xTBS+ 0.1% (v/v) Tween-20 (TBS-T)+10mM Na N3) was then incubated with the blot for overnight at room temperature (∼18–20hours). The blot was then washed twice in 1xTBS-T for 15 minutes, and once in 1×PBS for 15 minutes at room temperature. Absorbed antibody was then eluted by incubating with 0.2M glycine pH 2.5 at room temperature for 3 minutes. The pH of the eluted antibody solution was then neutralized by adding 0.09V of 1M Trisbase (pH unadjusted). NaN_3_ was then added to the solution to 10mM. The purified antibody was stored at 4°C.

### Western blot

For each sample, 5×10^6^ extracellular parasites were lysed by incubating in 1× SDS sample buffer (62.5 mM Tris pH 6.8, 2% (w/v) sodium deodeoyl sulfate, 10% (v/v) glycerol and ∼0.5 mg of bromophenol blue) containing 50 mM DTT at 100°C for 10 minutes. Western blot was performed as described in [38]. Affinity purified rat anti-TgMORN1 was diluted 1∶10 and mouse-anti-tubulin B-5-1-2 (Cat# T6074, Sigma, U.S.A) was diluted 1∶4,000 in TBS-T containing 0.5% (v/v) blocking buffer (Cat# 1152709001, Roche). Goat anti-rat IgG HRP (Cat# NA935V, GE Healthcare, United Kingdom) was diluted 1∶1000, and goat anti-mouse/rabbit HRP (Cat# 1152709001, Roche) were diluted 1∶20,000 in TBS-T containing 0.5% blocking buffer.

### Immunofluoresence

Intracellular parasites were fixed with 3.7% formaldehyde in 1×PBS for 15 minutes, permeabilized with 0.25 or 0.5% TX-100 in 1×PBS for 15 minutes, then blocked with 1 or 3% BSA in 1×PBS (blocking buffer) for 30 minutes at room temperature. The cells were then incubated in primary and subsequently secondary antibody solutions (diluted in blocking buffer) for 60 minutes each. Primary antibody dilutions were as follows: mouse anti-IMC1, 1∶1000 (A kind gift from Dr. Gary Ward, University of Vermont); rabbit anti-ACP (Kind gifts from Dr. Manami Nishi at McGill University and Dr. Dhanasekaran Shanmugam at University of Pennsylvania) 1∶500 or 1∶10; affinity purified rat anti-TgMORN1, 1∶10. Secondary antibody dilutions were as follows: goat anti-mouse Alexa 488(Cat# A11029, Molecular Probes-Invitrogen), 1∶2000; goat anti-mouse Alexa 568 (Cat# A11031, Molecular Probes-Invitrogen), 1∶2000; goat-anti-rat Cy3 (Cat#112-165-167, Jackson ImmunoResearch), 1∶500; goat-anti-rat Alexa 488 (Cat# A11006, Molecular Probes-Invitrogen), 1∶2000; goat anti-mouse Cy3 (Cat# 115-165-166, Jackson ImmunoResearch), 1∶500; goat anti-rabbit Alexa488 (Cat# A11034, Molecular Probes-Invitrogen), 1∶1000; goat anti-rabbit Cy3 (Cat# 111-165-144, Jackson ImmunoResearch), 1∶500; donkey anti-rabbit Alexa 488 (Cat# A21206, Molecular Probes-Invitrogen), 1∶1000; donkey anti-mouse Alexa 594 (Cat# A21203, Molecular Probes-Invitrogen), 1∶1000.

### Light microscopy

3D image stacks were collected at room temperature at z-increments of 0.3 µm on an Applied Precision Delta Vision imaging station constructed on an Olympus IX-70 inverted microscope base. A 100× oil immersion lens (NA = 1.4) and immersion oil at refractive index 1.518 were used for all the imaging. Deconvolved images were computed using the point-spread functions and software supplied by the manufacturer. All fluorescent images were maximum intensity projections of deconvolved 3D stacks unless otherwise stated. The brightness and contrast of images used in the final figures were optimized for color prints.

### Measuring the width of basal IMC1 gap in parental, *ΔTgMORN1* and *ΔTgMORN1/eGFP-TgMORN1* parasites

Measurement was performed on parasites in vacuoles containing one or two parasites. Specifically, parasites were labeled with IMC1 antibody as described above. 3-D stacks were acquired as described above, and images focused at the mid-section of the parasite were used for measuring the width of basal IMC1 gap in the parasites using Softworx (Applied Precisions, Inc.).

### Invasion and motility (trail) assays

Invasion assays were performed as previously described [43] with the following modifications. 1×10^7^ extracellular parental, *ΔTgMORN1* or *ΔTgMORN1/eGFP-TgMORN1* parasites were added to confluent HFF monolayers and incubated at 37°C for 1 hour. Cells were fixed in 3.1% formaldehyde and 0.06% glutaraldehyde (diluted in PBS) for 15 minutes at room temperature. Extracellular parasites were labeled with mouse anti-SAG1 antibody (Cat #11-132, Argene, North Massapequa, NY, diluted 1∶500) and visualized with goat anti-mouse Alexa 568 (1∶1,000). Cells were then permeabilized with 0.25% TX-100 diluted in PBS for 15 minutes at room temperature and intracellular and extracellular parasites were labeled with mouse anti-SAG1 and visualized with goat anti-mouse Alexa 488 (1∶1000). All antibody incubations were performed for 30 minutes. The number of invaded (green only) parasites was calculated by subtracting the number of extracelluar (dual-labeled) parasites from the total number of parasites on the coverslip. Images were taken from 6 randomly chosen fields at 10× magnification and counting was performed using MetaMorph® software. Results were from three independent experiments. Motility assays were performed as previously described [13].

### Cytokinesis and Intracellular replication assay

Equal numbers of parental, *ΔTgMORN1* or *ΔTgMORN1/eGFP-TgMORN1* parasites were added to confluent HFF monolayers and grown for 12, 18, and 24 hours. Immunofluoresence assay with rat anti-TgDIP13 diluted 1∶400 (for visualizing the apical complex), mouse anti-IMC1 diluted 1∶500, and DAPI diluted to 1µg/ml was performed as described above. To assess the effect of TgMORN1 deficiency on cytokinesis, the number of vacuoles containing at least one parasite displaying cytokinesis defects was counted for a total of 200 vacuoles per time point in each of 3 independent experiments. The counting was restricted to the vacuoles with fewer than 16 parasites, because in larger parasitophorous vacuoles *ΔTgMORN1* parasites were too disorganized for assessing the level of cytokinesis defect accurately.

Replication assays were performed as previously described [44]. The number of parasites/vacuole in 200 vacuoles per time point (18, 24, 30, and 40 hours) was counted in each of 3 independent experiments. Parasites that failed cytokinesis were counted as two. For the 40-hour time point, vacuoles containing one and two parasites were not included in the counting, as they were most likely secondary vacuoles formed by parasites that egressed from large primary vacuoles and reinvaded.

### Apicoplast segregation analysis

To analyze apicoplast segregation, parental, *ΔTgMORN1* and *ΔTgMORN1/eGFP-TgMORN1* parasites were grown for 20–24 hours and immunofluoresence was performed using an anti-ACP antibody as described above. For parental and *ΔTgMORN1/eGFP-TgMORN1* parasites, total 50 vacuoles were counted. For *ΔTgMORN1* parasites, total 250 vacuoles were counted (from 5 independent experiments; 50 vacuoles per experiment) and vacuoles were scored as apicoplast positive (*i.e.* all parasites in the vacuole contain apicoplast), apicoplast negative (*i.e.* none of the parasites in the vacuole contains apicoplast) or mixed (*i.e.* vacuoles contains both apicoplast positive and negative parasites).

### Plaque assay

Equal numbers of parental, *ΔTgMORN1*, and *ΔTgMORN1/eGFP-TgMORN1* parasites were allowed to infect and grow in fully confluent HFF for 11 days. The cultures were then fixed and permeablized in cold methanol (−20°C) for 15 minutes and stained with Coomassie® Brilliant Blue G-250 dye (BioRad, Catalog#: 500-0006) at room temperature for 2–3 hours, then 4°C overnight before scanning.

### Assay for virulence in mice

Freshly lysed out tachyzoites were filtered (3 µm), spun down and parasite pellet was resuspended in PBS. Parental (10^3^ or 10^4^), *ΔTgMORN1* (10^3^, 10^4^, 2×10^4^ or 10^5^) or *ΔTgMORN1/eGFP-TgMORN1* (10^3^ or 10^4^) tachyzoites (in 0.1 ml of PBS) were injected intraperitoneally (IP) into 6–8 week old CD1 outbred female mice (total 8 groups at n = 4; Charles River, QC). After 21 days, surviving mice “immunized” with 10^3^ or 10^4^ *ΔTgMORN1* parasites were challenged IP with 10,000 wild type RH tachyzoites.

### List of Tigr_final numbers for genes and proteins mentioned in the text

TgMORN1 (583.m05359); TgCentrin2 (50.m03356); TgIMC1 (44.m00004); *T. gondii* Acyl Carrier Protein (TgACP; 55.m00019); *T. gondii* α1 –tubulin (TgTubA1; 583.m00022); *T. gondii* dynein light chain (TgDLC; 41.m01383).

## Supporting Information

Figure S1 The arrangement of cortical microtubules in the parental and the TgMORN1 knock-out parasites. Surface optical sections of the parental and the TgMORN1 knock-out parasites expressing eGFP-TgTubA1, which indicate that the arrangement of cortical microtubules around the TgMORN1 knock-out parasite cortex appears to be normal. Arrows: cortical microtubules. Arrowheads: conoid.
(0.13 MB TIF)

Table S1 Sequences of the 521 bp DNA fragment and primers used for PCR amplification for constructing the plasmids listed in the left column. For primers, restriction sites are shown in lower case.
(0.05 MB PDF)
