# Supplementary figures and images for: TgMORN1 Is a Key Organizer for the Basal Complex of Toxoplasma gondii
Source: PLoS Pathog. 2010 Feb 5;6(2):e1000754. doi: 10.1371/journal.ppat.1000754 (PMC2816694; doi:10.1371/journal.ppat.1000754)

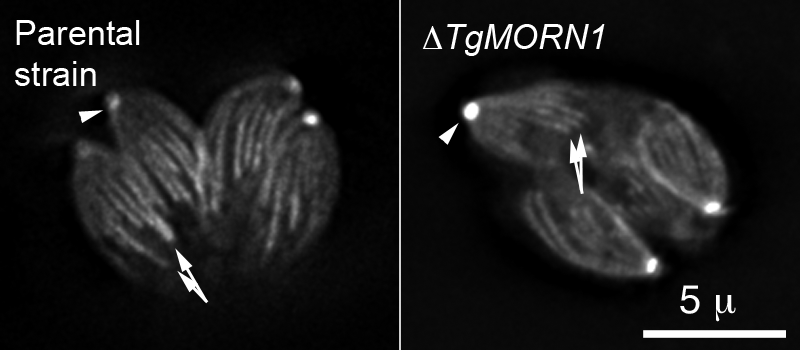

Supplement: Figure S1 — The arrangement of cortical microtubules in the parental and the TgMORN1 knock-out parasites. Surface optical sections of the parental and the TgMORN1 knock-out parasites expressing eGFP-TgTubA1, which indicate that the arrangement of cortical microtubules around the TgMORN1 knock-out parasite cortex appears to be normal. Arrows: cortical microtubules. Arrowheads: conoid. (0.13 MB TIF) [file ppat.1000754.s001.tif]
