# Supplementary material for: TgMORN1 Is a Key Organizer for the Basal Complex of Toxoplasma gondii
Source: PLoS Pathog. 2010 Feb 5;6(2):e1000754. doi: 10.1371/journal.ppat.1000754 (PMC2816694; doi:10.1371/journal.ppat.1000754)
Supplement: Table S1 — Sequences of the 521 bp DNA fragment and primers used for PCR amplification for constructing the plasmids listed in the left column. For primers, restriction sites are shown in lower case. (0.05 MB PDF) [file ppat.1000754.s002.pdf]

PTKO2_II	GGTACCCTCGAGGATATCTACGAATTCATAACTTCG<br>TATAGCATACATTATACGAAGTTATAGATCTGTTTA<br>AACGCGATCGCGGTCCGAGGCCTGACTACGACGAAA<br>GTGATGCGCAGGCTGGAAAGCCGCTGAAGGGAGAAG<br>TCTACAAAGCCGATCAGTGAAAAATGTGTGGGGAGG<br>TGGTCTTGTTGCAGGAATGCAATGGTGTTAAGCATC<br>GTGTTCGAATGCAGTGCGTGTATCAGTTGTGCGCGG<br>AAGGACACTGCTTCAATGTTAAGAACCTGTTTTCTC<br>CGTAGAGAGGACCAAAAGACGATTGCAAAACTGGTA<br>TGTACGCAATAGCCCAATGCCGGACGTCAGTTGGTT<br>GTATGTGACGCTCCCAGATGTCATATGCCTTGTGAG<br>TGTGTCTGGGATGCAAGTTTTGGTGTGCGTTGATT<br>TCGCCAGCTTATGACAGTGGCAGACGAATTATTGAC<br>ATGATACAAGGACGCAGAAAGGAACAAACACCGTAG<br>TTCCAGTCGACACTAGT
PTKO2_II_3'UTR	HK180 ATCGgctagcACGATGTCAGTTTTTCTGCGAGC<br>HK181 ATCGgggcccCCCATTTTCTAATTTCAGAATACG
PTKO2_II_TgMORN1_5_3'UTR.	HK182<br>ACTGggtaccGACCGAGCTTGAGATGAACTTATTG<br>HK 183<br>ACTGgaattcGTAAGGAAAAACGGGGAATGAAGTG
LoxP-TgMORN1-HXGPRT-LoxP	HK191<br>ATGCagatctCGTTGTCCACCATGGAGAGCTGCCA<br>CGCGTACCACGGAC (Underlined: Kozak sequence)<br>HK193<br>ACTGaggcctTTACAAGTCGACATTGAGCCATGG
ptub-Cre-GFP	HK223<br>ATCGgctagcAAAATGGCCAATTTACTGACCGTACAC<br>HK224<br>ATCGcttaagTTACTTGTACAGCTCGTCCATGCC

Table S1. Sequences of the 521 bp DNA fragment and primers used for PCR amplification for constructing the plasmids listed in the left column. For primers, restriction sites are shown in lower case.
